# Insulin, CCAAT/Enhancer-Binding Proteins and Lactate Regulate the Human 11β-Hydroxysteroid Dehydrogenase Type 2 Gene Expression in Colon Cancer Cell Lines

**DOI:** 10.1371/journal.pone.0105354

**Published:** 2014-08-18

**Authors:** Thomas Andrieu, Pierre Fustier, Rasoul Alikhani-Koupaei, Irena D. Ignatova, Andreas Guettinger, Felix J. Frey, Brigitte M. Frey

**Affiliations:** Department of Nephrology & Hypertension and Clinical Pharmacology and Department of Clinical Research, University Hospital of Berne, Berne, Switzerland; University Medical Center Utrecht, Netherlands

## Abstract

11β-Hydroxysteroid dehydrogenases (11beta-HSD) modulate mineralocorticoid receptor transactivation by glucocorticoids and regulate access to the glucocorticoid receptor. The isozyme 11beta-HSD2 is selectively expressed in mineralocorticoid target tissues and its activity is reduced in various disease states with abnormal sodium retention and hypertension, including the apparent mineralocorticoid excess. As 50% of patients with essential hypertension are insulin resistant and hyperinsulinemic, we hypothesized that insulin downregulates the 11beta-HSD2 activity. In the present study we show that insulin reduced the 11beta-HSD2 activity in cancer colon cell lines (HCT116, SW620 and HT-29) at the transcriptional level, in a time and dose dependent manner. The downregulation was reversible and required new protein synthesis. Pathway analysis using mRNA profiling revealed that insulin treatment modified the expression of the transcription factor family C/EBPs (CCAAT/enhancer-binding proteins) but also of glycolysis related enzymes. Western blot and real time PCR confirmed an upregulation of C/EBP beta isoforms (LAP and LIP) with a more pronounced increase in the inhibitory isoform LIP. EMSA and reporter gene assays demonstrated the role of C/EBP beta isoforms in HSD11B2 gene expression regulation. In addition, secretion of lactate, a byproduct of glycolysis, was shown to mediate insulin-dependent HSD11B2 downregulation. In summary, we demonstrate that insulin downregulates HSD11B2 through increased LIP expression and augmented lactate secretion. Such mechanisms are of interest and potential significance for sodium reabsorption in the colon.

## Introduction

The mineralocorticoid receptor (MR) is essential for renal sodium handling in epithelial tissues such as colon and kidney and for blood pressure control in humans. The physiological ligand of the MR is aldosterone [1]. Another adrenal steroid, cortisol, exhibits a similar affinity and transactivation potential for the MR as aldosterone. Serum concentrations of cortisol are 100 to 1000 fold higher than aldosterone. The mechanism allowing aldosterone to be the preferred ligand for the MR *in vivo*, despite the higher concentrations of cortisol is an enzyme which inactivates cortisol, specifically in MR expressing cells [2]. This enzyme, 11β-hydroxysteroid dehydrogenase type 2 (11beta-HSD2) is encoded by the HSD11B2 gene and converts biologically active cortisol into cortisone, a steroid with negligible affinity and activation potential for the MR [3]. Thus, a reduced activity of 11beta-HSD2 causes cortisol-mediated MR activation, leading to renal sodium retention, suppression of renin and a salt-sensitive increase in blood pressure [4], [5].

Many patients with type 2 diabetes have low renin activity in plasma and are salt-sensitive [6]–[8]. Furthermore, we recently observed an association of salt-sensitivity and reduced activity of 11beta-HSD2 in offspring of type 2 diabetic patients [9]. Thus, it is reasonable to speculate that insulin downregulates HSD11B2, and by this mechanism, causes cortisol-mediated renal or colonic sodium retention with consequent renin suppression.

It has been shown that that insulin and hyperinsulinemia regulate the family of transcription factors CCAAT/enhancer binding proteins (C/EBPs) [10], [11] and that members of this family control the transcription of HSD11B1 [12]–[14]. The analysis of the promoter of the HSD11B2 gene by the transcription factor database (TRANSFAC) program revealed several putative binding sites for C/EBPs at positions −4362 bp, −1985 bp, −177 bp and at −198 bp from the transcriptional starting site of human HSD11B2 gene. Therefore, we hypothesized that insulin down-regulates HSD11B2 through C/EBPs.

## Material and Methods

### Reagents and supplies

Cell culture material was from Becton Dickinson Labware (Basel, Switzerland) and Corning (Bodenheim, Germany). Dulbecco's Modified Eagle's Medium (DMEM) and Mc Coy's were purchased from Sigma Chemicals (Buchs, Switzerland). Fetal bovine serum (FBS) was from Biochrom AG (Berlin, Germany). Insulin, cycloheximide (CHX), PD098059, Sodium Dichloroacetate (DCA), Sodium L-lactate and 5,6-dichlorobenzimidazole 1β-D-ribofuranoside (DRB) were purchased from Sigma-Aldrich (Fluka AG, Buchs, Switzerland). The following cell lines were purchased from ATCC (Manassas, VA): human colon carcinoma cell line HT-29 (accession number: HTB-38), SW-620 (CLL-227), HCT116 (CCL-247) and JEG-3 (HTB-36). Akt Inhibitor VIII was from Calbiochem (Merck, Zug, Switzerland). Adenosine 5′-triphosphate [gamma-^32^P] ([gamma^32^P] ATP) was from Perkin Elmer (Maanstraat, The Netherlands).

### Cell Cultures

HCT116, SW-620 and HT-29 were grown in DMEM supplemented with 10% FBS, 2 mmol/L glutamate, 100 U/ml penicillin, and 100 µg/ml streptomycin. The cells were maintained at 37°C in humidified 5% CO2-95% air. All cell lines were plated in cell culture dishes and grown in DMEM 10% FBS to confluence. Cells were incubated in DMEM supplemented with 0.3% FBS during insulin and DCA treatment. Cells were treated 48 h with DCA and 24 h with insulin. Cells were incubated with 10% FBS during lactate treatment after 24 h synchronization in DMEM 0.3% FBS.

### RNA preparation and expression level

Total RNA was isolated using RNeasy Mini Kit (Qiagen AG, Basel, Switzerland) according to the manufacturer's protocol. Total RNA (1 µg) was used for the synthesis of first strand cDNA using the Improm-II Reverse Transcriptase (RT) in RT buffer (Promega Catalys AG, Wallisellen, Switzerland) according to the manufacturer's protocol. Expression of specific mRNA was determined by quantitative real-time RT-PCR (qRT-PCR) on an ABI PRISM 7000 Sequence Detection System (Applied Biosystems, Foster City, CA). Multiplex PCR was performed according to the manufacturer's protocol (Applied Biosystems, Foster City, CA). Assays-on-Demand (Gene Expression Assay Mix) were eukaryotic 18S rRNA endogenous control (4310893E), HSD11B2 (Hs00388669_m1), CCAAT/enhancer binding protein (C/EBP) alpha (Hs00269972_s1), C/EBP beta (Hs00270923_s1) and C/EBP delta (Hs00270931_s1). Relative gene expression was determined using the comparative CT (threshold cycle) method, which consists of the normalization of the number of target gene copies to an endogenous reference gene (18S rRNA), designated as calibrator. The level of HSD11B2, C/EBP alpha, C/EBP beta and C/EBP delta mRNA expression of each of the treated cells was normalized to the result obtained from untreated cells. The amount of target normalized to the 18S rRNA endogenous reference is given by the formula: 2^−ΔΔCT^. To confirm the reproducibility of mRNA determination, a minimum of 3 independent total RNA extractions were performed. Each reverse-transcriptase polymerase chain reaction (RT-PCR) assay was analyzed in triplicate and expressed as mean +/− SD.

### Measurement of 11beta-HSD2 activity

Cells were cultured in 6-well plates at a density of 0.5×10^6^ cells/well. After treatment, culture medium was removed and cells were incubated for 45 min in 1 ml medium containing 2 µCi of [1,2,6,7-^3^H] Cortisol (60–80Ci/mmol, Amersham, Buckinghamshire, UK) in 6-well plates. After incubation the reaction was stopped and the steroids were extracted by the addition of three volumes of ethyl acetate. After centrifugation, the organic phase was removed and evaporated at room temperature. The residue was reconstituted in 30 µl of stop solution (2 mM cortisol and 2 mM cortisone in methanol). Ten microliters were applied to silica–coated TLC plates (G-25, UV254, Macherey-Nagel, Oensingen, Switzerland) and resolved using chloroform: ethanol (9∶1). Steroids were visualized under ultraviolet light and were scraped into scintillation fluid. The radioactivity was measured using a Packard 2000CA Tri-Carb Liquid Scintillation Analyzer (Packard Instrument Co, Downers Grove, IL). Experiments were carried out under non-substrate limiting conditions, where metabolism was always less than 40%. Specific activity was expressed as picomoles (pmoles) per micrograms of protein per hour. The experimental results were calculated by expressing the conversion rates of cortisol to cortisone in the presence of insulin, as a percentage of that in the corresponding control in absence of insulin [15].

### Western blot analysis

Protein extraction and Western blot analyses were performed as reported earlier [16]. Briefly, nuclear extracts were isolated with the CelLytic NuCLEAR Extraction protocol (Sigma Chemicals, Buchs, Switzerland). For Western blot analysis, total protein (100 µg) and nuclear extracts (60 µg) were loaded on a denaturing 10% polyacrylamide gel. The membrane was blocked overnight and incubated with rabbit polyclonal antibody for 11beta-HSD2 (H-145,1∶500), C/EBP alpha (sc-61X, 1∶5000), C/EBP beta (sc-150X, 1∶5000), β-actin (sc-1616R, 1∶500) or HDAC1 (sc-7872, 1∶500) (Santa Cruz Biotechnology, Santa Cruz, CA). Washed nitrocellulose membranes were incubated with a goat anti-rabbit IgG horseradish peroxidase conjugate (sc-2004, Santa Cruz Biotechnology, Santa Cruz, CA) and developed using enhanced chemiluminescence (ECL) reagent (Amersham, Buckinghamshire, UK). Densitometry of exposed films was performed and the level of protein expressed as arbitrary units.

For detection of IGF1 and insulin receptors cells were either untreated or treated with 100 nM insulin for 24 h and lysed in RIPA buffer containing 1 mM sodium orthovanadate, 2 µg/ml aprotinin, 1 µg/ml leupeptin, 1 mM phenylmethanesulfonyl fluoride. The lysates were incubated with either IGFR or Insulin Receptor antibodies (3027, 3025, Cell Signaling) at 4°C overnight. In the morning, the samples were incubated with Protein A/G Plus Agarose (Santa Cruz Biotechnology) for 1 h at 40°C. The beads were washed, boiled in SDS loading buffer and proteins were separated by SDS-PAGE.

### *De novo* protein synthesis

HT-29 cells were cultured as outlined above and pretreated with the protein synthesis (or translational elongation) inhibitor, CHX (10 µM) for 1 h before the addition of insulin (10^−7^ M). At the end of the 24 h treatment, cells were harvested for RNA isolation and qRT-PCR analysis.

### HSD11B2 mRNA stability

HT-29 cells were cultured as outlined above and treated with insulin (10^−7^ M) for 12 h. Transcription was stopped with DRB (25 µM) and cells were harvested at discrete times (0–12 h) for RNA isolation and qRT-PCR analysis.

### Small interfering RNA (siRNA) experiments

HT-29 cells were transiently transfected using Lipofectamine 2000 (Invitrogen, Carlsbad, CA, USA) following the manufacturer's recommendations. The transfection mixture was removed after 24 h incubation. The cells were further incubated under normal growth conditions for another 24 h before mRNA extraction. The siRNA duplexes for C/EBP alpha or C/EBP beta (Qiagen AG, Basel, Switzerland) and a negative control siRNA (Invitrogen, Carlsbad, CA, USA) were used for transfection at a final concentration of 50 nM.

### Electrophoretic mobility shift assay (EMSA) and nuclear extract preparation

Around five million of adherent cells were detached with 3 ml of PBS on ice and were pelleted for 5 min at 900 g. Pellets were stored at −80°C until protein extraction. Nuclear extract preparation and EMSA were performed as previously described [17], [18]. The protein yield was determined by the Bradford method. EMSA probes were generated by annealing complementary single-stranded oligonucleotides and labeled with [gamma^32^P] ATP and T4 polynucleotide kinase. Specific binding was competed with unlabeled oligonucleotides which sequence is recognized by the C/EBP factors at a 100X-molar excess (5′-tgcag**attgcgcaat**ctgca-3′; the nucleotide motifs of interest are bold-faced). The binding reactions were carried out in 10 µl of buffer [20 mM HEPES, pH 7.5; 35 mM NaCl; 60 mM KCl; 0.01% NP 40; 2 mM DTT; 0.1 mg/ml BSA; 4% ficoll] containing 1.75 pmol of labeled probe, 4 µg nuclear proteins and 1 µg poly (dI-dC). Mixtures were incubated at 4°C for 20 min in presence or absence of unlabeled competitor. DNA-protein complexes were separated on a 5% polyacrylamide gel in 0.5× Tris-borate-EDTA buffer for 90 min at 140 V. Gels were dried 2 h at 80°C and analyzed on a PhosphoImager Cyclone (Packard).

### Chromatin immunoprecipitation

ChIP assays were performed according to the instruction of Upstate Biotechnology Inc as previously reported [18]. Purified DNA fragments were amplified with PCR primers to detect a 210 bp fragment containing the −177C/EBP, -198C/EBP sites within the HSD11B2 promoter (forward: 5′-GCAACTTTGGGACTTTGTTCCGGC-3′; reverse: 5′-AGAGGGACACTCGCTTTCTCTGCT-3′).

### qRT-PCR analysis using human diabetes RT^2^ Profiler PCR Arrays

The RT^2^ Profiler PCR Arrays PAHS-30C (SA Biosciences, MD, USA) was designed to analyze 84 genes related to human insulin signaling pathway. The RT-PCR was carried out using an ABI PRISM 7000 Sequence Detection System (Applied Biosystems, Foster City, CA). HT-29 cells were treated for 24 h with insulin (10^−7^ M). Total RNA (1 µg) was used as template to synthesize cDNA with the RT^2^ First Strand kit (SABiosciences). The PCR cycle condition was as follows: 95°C for 10 min, followed by 40 cycles of 95°C for 15 s, 60°C for 60 s. At the end of PCR cycling steps, data for each sample were displayed as a melting curve. The ABI SDS software (Applied Biosystems) was used to determine a critical threshold (Ct), which was the cycle number where the linear phase for each sample crossed the threshold level. Beta-2-microglobulin was used as housekeeping gene. The expression of HSD11B2 for the 3 experiments concerned was monitored in parallel by real time PCR which confirming significant downregulation by insulin. Records were deposed in the GEO data base with accession number GSE51677.

### Transient transfection and reporter gene assay

Transfections were performed with FuGENE HD transfection reagent (Roche, Rotkreuz, Switzerland) using 3 ml of solution for 1 mg of plasmid. The vector pCMV-hRL (*Renilla reniformis luciferase*) (Promega Catalys AG, Wallisellen, Switzerland) was used for normalization of transfection efficiency. The construct p4.5 kb-HSD11B2 was a generous gift from de. K. Yang [19]. The p0.2 kb-HSD11B2 plasmid construct was described previously [18]. For expression of transcription factors, various amounts of the vectors pCMV-LIP and pCMV-LAP, a generous gift from U. Schibler [20], were added to the DNA mixture. After 6 h the transfection medium was replaced with normal growth medium for 18 h. Thereafter cells were lysed and luciferase activities were detected with the Dual-Luciferase Reporter Assay System (Promega Catalys AG, Wallisellen, Switzerland) and MediatorsPhL Luminometer (Mediators Diagnostic Systems, Vienna, Austria). Firefly luciferase activity was expressed relative to Renilla luciferase to account for differences in transfection efficiency. When a CMV-LacZ control vector was transfected, Dual-Light system (Applied Biosystems, Foster City, CA) was used to determine the luciferase activity. Transfections were confirmed by multiple independent experiments.

### Site-directed mutagenesis

HSD11B2 promoter mutants were generated in the p4.5 kb-HSD11B2 and p0.2 kb-HSD11B2 constructs using QuikChange II XL site-directed mutagenesis kit (Stratagene, Basel, Switzerland). The following primers were used: 5′-GTGGAACTTGAGAGCT**C**GAGCA**G**TTCCCTTCACCTCTGG-3′ and 5′-CCAGAGGTGAAGGGAA**C**TGCTC**G**AGCTCTCAAGTTCCAC-3′ for -4362 C/EBP and 5′-CTCGAGCGCAGC**CG**CT**CCA**GGACTTTGTTCCGGCTTTTTC-3′ and 5′- GAAAAAGCCGGAACAAAGTCC**TGG**AG**CG**GCTGCGCTCGAG- 3′ for -198 C/EBP. Underlined and bolded letters represent mutated bases.

### Bioinformatics and statistics

Data are expressed as mean +/− SD of triplicate samples of a representative experiment repeated at least three times. Statistical analysis was performed using the Student's *t* test or ANOVA analyses and was followed by a contrast test with Tukey error protection. Differences were considered significant at *p*<0.05, *p<0.05, **p<0.01, ***p<0.001. Transcription factor binding sites were analyzed with the Match program.

## Results

### Sustained insulin treatment decreases 11beta-HSD2 activity and HSD11B2 gene expression in colonic cancer cell lines

**Figure 1:**
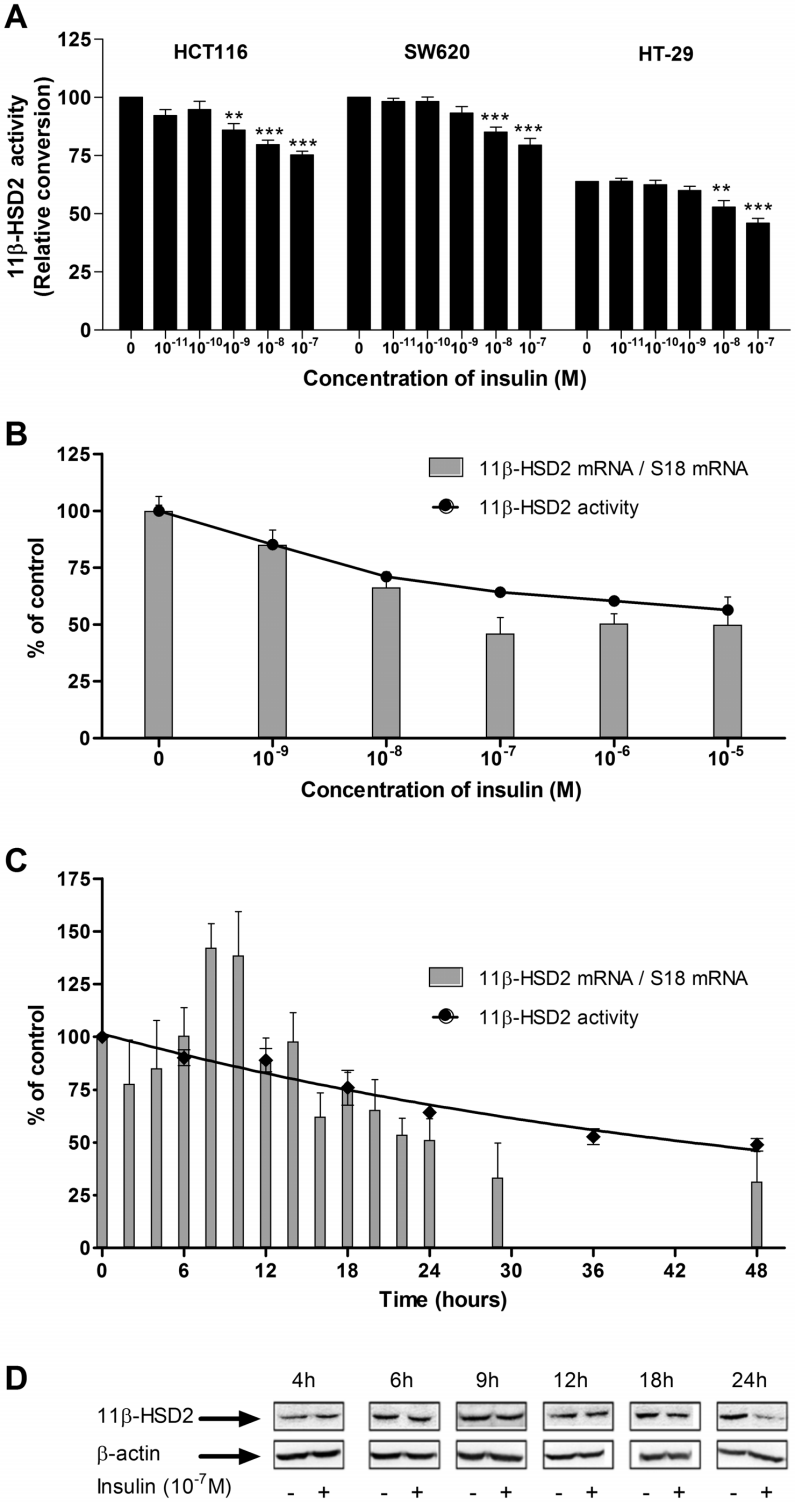
Sustained insulin treatment diminished the 11beta-HSD2 expression and activity in HT-29 cells. (*A*) 11beta-HSD2 activity was measured by ^3^H-cortisol/cortisone conversion assay in colonic cell lines 24 h after incubation with insulin (10^−11^–10^−7^ M). The activity measured for HCT116 in absence of insulin was set as 100%. (*B*) Dose-response effect of insulin (10^−9^–10^−5^ M) on HSD11B2 mRNA (gray bars) and activity (curve) in HT-29 cells treated for 24 h. (*C*) Time-dependent effect of insulin (10^−7^ M) on HSD11B2 mRNA (gray bars) and activity (curve) in HT-29 cells. (*D*) Time-dependent effect of insulin (10^−7^ M) on 11beta-HSD2 protein level.

Regulation of enzyme activity by insulin was examined in HSD11B2 expressing [16]–[18], [21] human colonic cell lines (HCT116, SW620 and HT-29) (Fig. 1A). Cells were incubated for 24 h with insulin (10^−11^ M-10^−7^ M) in cell culture medium containing 0.3% FBS. Insulin caused a dose response decrease in 11beta-HSD2 activity in all tested colonic cell lines with a significant reduction at 10^−9^ M in HCT116 cell line (*p*<0.05). This effect was not restricted to colonic cell lines since similar results were obtained with HSD11B2 expressing [19] JEG-3 cells (Fig. S1). Due to the robust response (≈30% reduction), we further characterized the molecular mechanisms in HT-29 cells.

### Sustained insulin treatment decreases HSD11B2 gene expression, activity and protein in HT-29 cells in a dose- and time-dependent manner

We next confirmed whether insulin-reduced 11beta-HSD2 activity coincides with its gene and protein expression (Fig. 1A). Increasing concentrations of insulin ranging from 10^−9^ to 10^−5^ M caused a concentration-dependent decrease in HSD11B2 mRNA levels 24 h after treatment (Fig. 1B). A maximal effect was observed at concentration of 10^−7^ M, where HSD11B2 mRNA was lowered by 50% (*p*<0.05) (Fig. 1B); and the activity by 35% (*p*<0.05) (Fig. 1B). Thereafter, we investigated the time-dependent regulatory effect of insulin on HSD11B2 gene expression, activity, and protein level. As shown in Figure 1C, a time-dependent decrease in 11beta-HSD2 activity was observed with a significant reduction 12 h after treatment (*p*<0.05). Interestingly the HSD11B2 mRNA increased during the first 10 h and decreased thereafter. After 16 h the mRNA reached minimal levels and remained low up to 48 h (*p*<0.05) (Fig. 1C). In agreement, HSD11B2 protein was reduced by 18 h and 24 h of insulin treatment (Fig. 1D).

**Figure 2:**
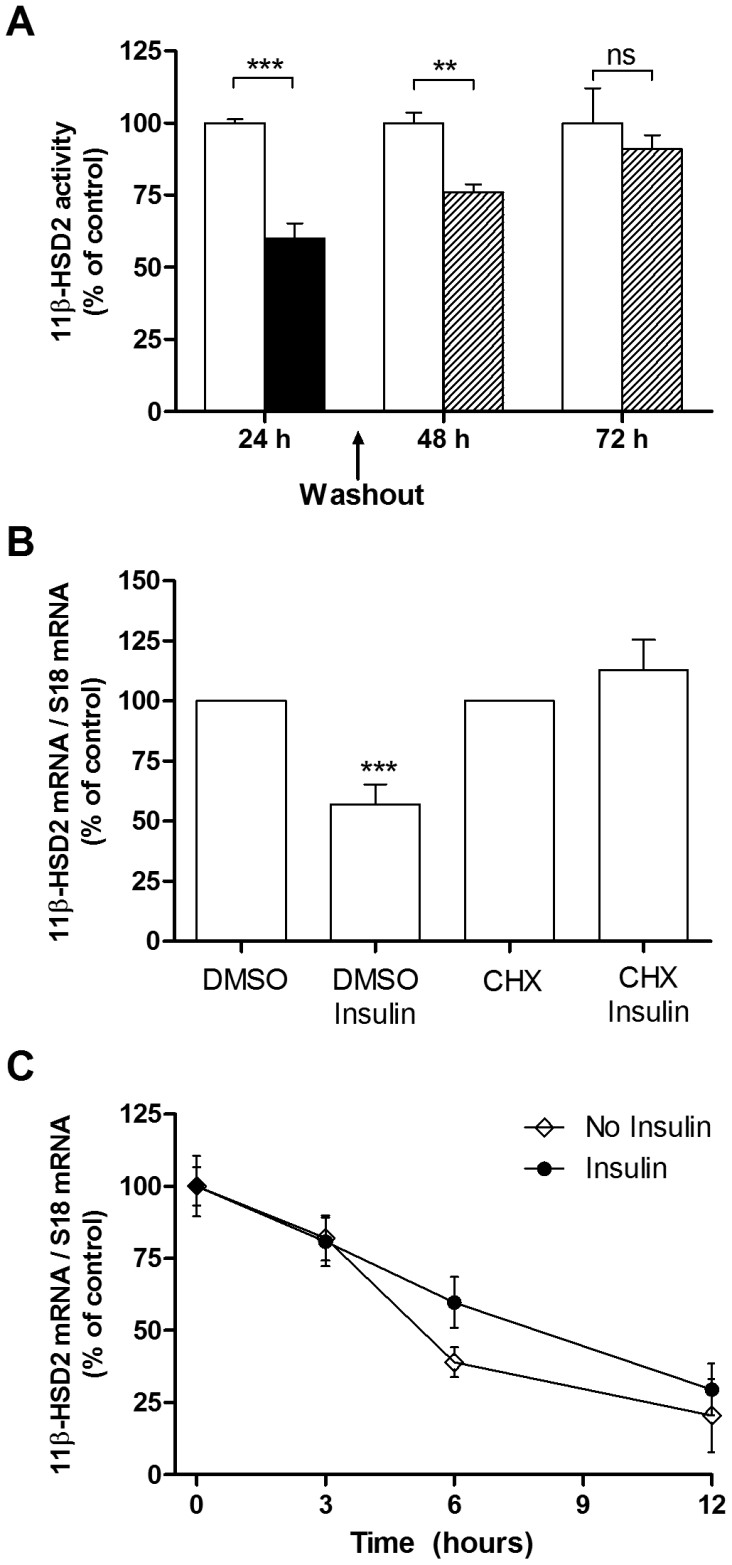
Insulin-dependent decrease of 11beta-HSD2 activity and mRNA is reversible but HSD11B2 half-life is not affected. (*A*) 11beta-HSD2 activity was measured in HT-29 cells in presence (black bars) and absence (white bars) of 10^−7^ M insulin for 24 h. The insulin effect was reversible on washout with PBS 24 h or 48 h of follow-up (hatched bars). (*B*) HSD11B2 expression was assessed by qRT-PCR in HT-29 pretreated with the protein synthesis inhibitor CHX (10 µM) for 1 h and treated with insulin (10^−7^ M) for 24 h. Each data point is expressed as a percentage of the control value. (*C*) HT-29 cells were pretreated with (filled circles) or without (filled rhombus) insulin (10^−7^ M) for 12 h. The cells were then treated with 25 µM of the mRNA synthesis inhibitor DRB, without or with insulin (10^−7^ M) (defined as time zero). At the indicated time points thereafter, total RNA was isolated, and the steady state level of HSD11B2 mRNA assessed. Each data point is expressed as a percentage of the maximum determined at time zero.

The downregulation of HSD11B2 was reversible by removing insulin from the medium 24 h after incubation. Indeed, 48 hours after the removal, HSD11B2 mRNA levels in control and insulin treated conditions were similar (Fig. 2A). Next, HT-29 cells were treated 24 h with insulin in the absence and presence of the protein synthesis inhibitor, cycloheximide (CHX). The effect of insulin in reducing HSD11B2 mRNA was abolished in the presence of CHX, indicating that *de novo* protein synthesis was required (Fig. 2B). To determine whether insulin reduces the HSD11B2 mRNA stability, we assessed the half-life of HSD11B2 mRNA by a standard mRNA decay assay using 25 µM DRB, an inhibitor of mRNA synthesis. As shown in Figure 2C, insulin did not alter the half-life of HSD11B2 mRNA.

### Insulin pathway analysis

**Figure 3:**
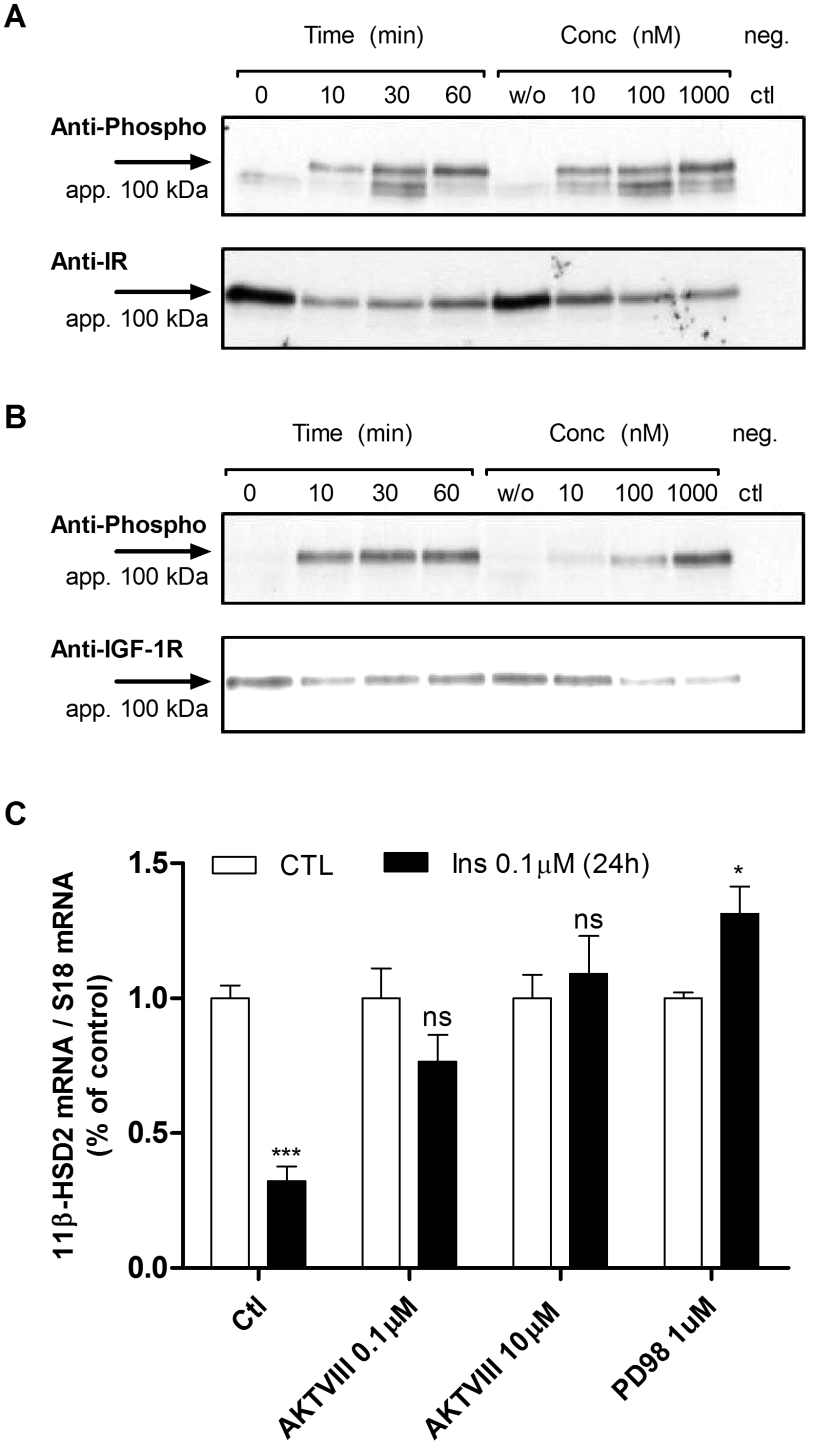
Insulin activates insulin and IGF-1 receptors and PI3K/AKT – MEK downstream pathways. (*A*) Expression and phosphorylation of insulin receptor by insulin in a time (0 to 60 min) and dose (0 to 1000 nM) dependent manner. (*B*) Expression and phosphorylation of IGF-1 receptor by insulin in a time and dose dependent manner. (*C*) HSD11B2 mRNA expression monitored by qRT-PCR in presence of insulin, AKT inhibitor (AKTVIII, 0.1 µM and 10 µM) or MEK inhibitor (PD098059, 1 µM).

In order to understand the molecular mechanism by which insulin down-regulates HSD11B2 we aimed to characterize the insulin pathway in HT-29. Western blot experiments demonstrated the expression and activation of IGF-1 (IGFI-R) and insulin receptors (IR) in a time and dose dependent manner (Figs. 3 A, B). Both receptors are phosphorylated within the first 10 min upon insulin treatment, while IR was more sensitive than IGFI-R to low doses of insulin (Figs. 3 A, B). The role of downstream kinases on insulin-dependent HSD11B2 repression was assessed using PD098059 and AKT VIII inhibitors. Figure 3C shows that both pathways, the MAPK/ERK and the PI3K pathway, mediated the insulin effect.

**Figure 4:**
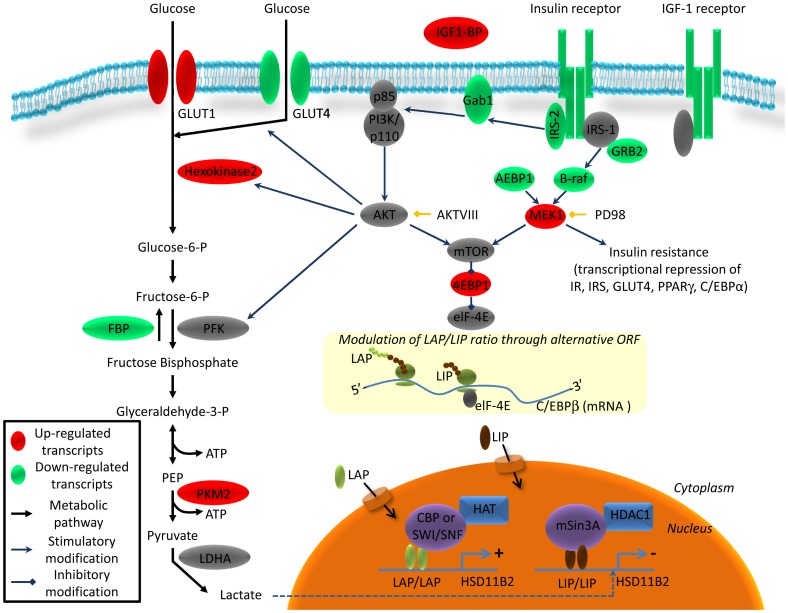
Schematic representation of the insulin pathway and its regulation by sustained insulin stimulation in HT-29. mRNAs were quantified 24 h after insulin (10^−7^ M) treatment using RT^2^ Profiler PCR Arrays PAHS-30C. Up-regulated transcripts are shown in red and down-regulated transcripts are shown in green.

Total mRNA of insulin treated HT-29 cells was extracted and subjected to RT^2^ profiling to quantify the expression of insulin pathway components. The Human Insulin Signaling Pathway RT^2^ Profiler PCR Array profiles the expression of 84 genes related to insulin-responsive genes. Twenty two genes differentially regulated in HT-29 cells after insulin treatment are reported in Table S1 and the pathways involved are depicted in the scheme of Figure 4. RT^2^ profiler revealed a characteristic pattern of insulin insensitivity, with reduced expression of insulin pathway components: IR, IGFI-R, insulin receptor substrate (IRS2) and insulin regulated glucose transporter (GLUT-4). Sustained insulin treatment also promoted glycolysis in HT-29 cells. While insulin regulated glucose transporter GLUT-4 expression was downregulated, GLUT-1 encoding messenger was increased, facilitating the import of glucose into the cells, independently of growth factor stimulation. Hexokinase 2, the enzyme which phosphorylates glucose to glucose-6-P, a rate limiting step of glycolysis, was up-regulated, along with pyruvate kinase 2 (PKM2), which converts PEP into pyruvate. In contrast, the enzyme which dephosphorylated fructose 1, 6 bisphosphate into fructose-6-phosphate and contributed to antagonizing glycolysis was downregulated.

### Effect of insulin on C/EBP alpha, C/EBP beta, and C/EBP delta mRNA levels

**Figure 5:**
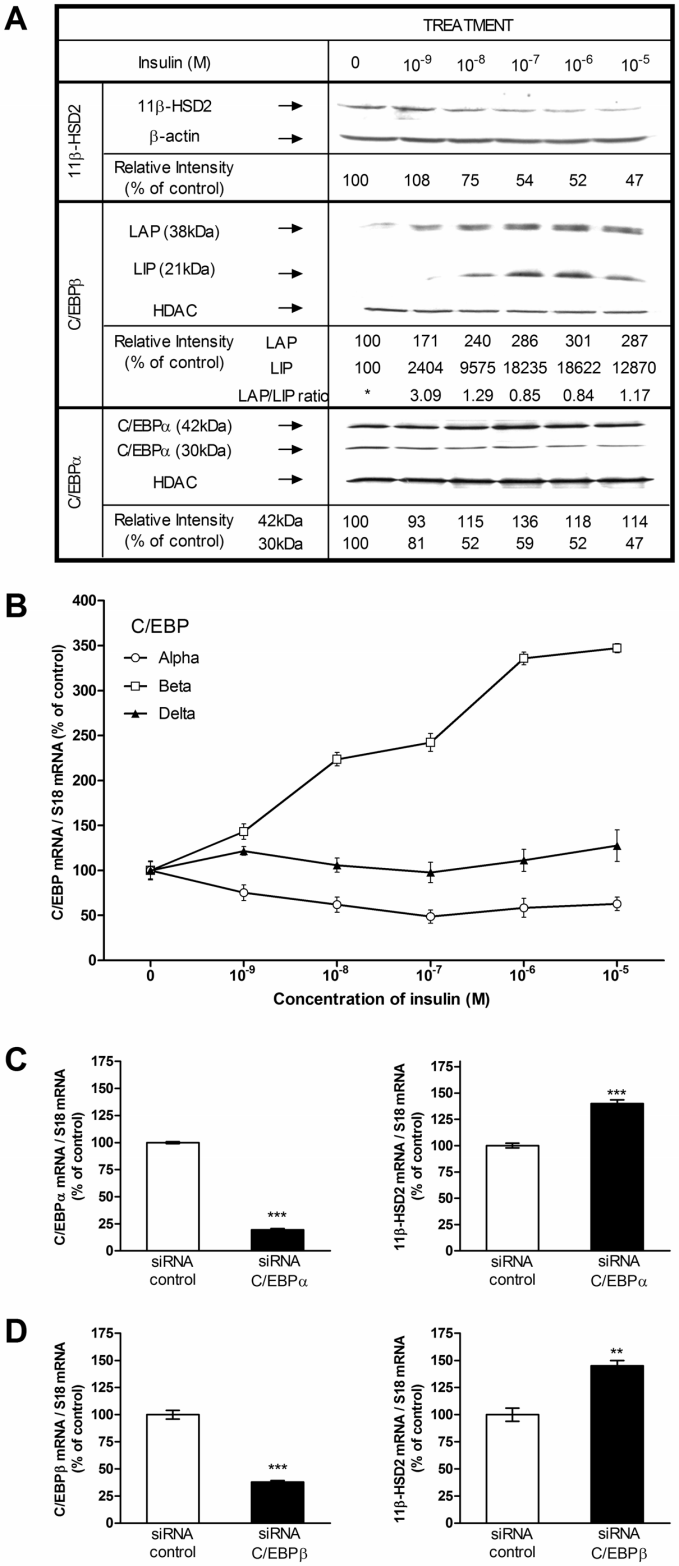
Insulin-dependent regulation of the 11beta-HSD2 protein level and role of the CCAAT/enhancer-binding protein (C/EBP) family. (*A*) Concentration-dependent effects of insulin on 11beta-HSD2, C/EBP alpha, and C/EBP beta protein levels. HT-29 cells were cultured for 24 h without and with increasing concentrations of insulin (10^−9^–10^−5^ M), then harvested for Western blotting to evaluate expression of 11beta-HSD2, C/EBP alpha, C/EBP beta. (*B*) Concentration-dependent effects of insulin on C/EBP alpha, C/EBP beta, and C/EBP delta mRNA expression. HT-29 cells were treated like in (A). The level of C/EBP alpha (open circles), C/EBP beta (open squares), and C/EBP delta (filled triangles) mRNA was measured using qRT-PCR with S18 as internal control. Expression levels in treated cells were normalized to untreated controls (100%). Representative data for at least three independent experiments. The relative intensity was determined by densitometric scanning. The ratio of relative densities of 11beta-HSD2 to beta-actin in cells cultured in the abscence of hormone was considered as 100% (control). The ratio of relative densities of nuclear extract proteins to HDAC in cells cultured without hormone was considered as 100% (control). * LIP was undetectable in the control samples, so the LAP/LIP ratio was not calculated. (*C, D*) Silencing of C/EBP alpha (*C*) and C/EBP beta (*D)* was performed using siRNA. The expression of C/EBP alpha, C/EBP beta (left panel) and HSD11B2 (right panel) mRNA was measured using qRT-PCR.

We present evidence in Figure 5B, that treatment of HT-29 cells with various concentrations of insulin (10^−9^–10^−5^ M) for 24 h caused a concentration-dependent increase in C/EBP beta mRNA expression. In contrast, insulin suppressed the expression of C/EBP alpha mRNA expression in a dose-dependent manner. At a concentration of 10^−7^ M, insulin decreased the C/EBP alpha mRNA by 51% (*p*<0.01), whereas C/EBP delta mRNA expression was unchanged. These results show that insulin-dependent reduction of HSD11B2 mRNA, correlates with the expression pattern of 2 out of 3 investigated members of the C/EBP family of transcription factors in HT-29 cells.

### Insulin-regulation of C/EBP alpha and C/EBP beta proteins

To investigate whether C/EBP alpha or C/EBP beta play a role in the insulin-dependent repression of HSD11B2 gene expression, the expression of C/EBP alpha and C/EBP beta in HT-29 cells were analyzed by Western blots (Fig. 5A). C/EBP alpha mRNA may lead to two polypeptides with a size of 42 kDa and 30 kDa [22], [23] while C/EBP beta might evolve to an activating or an inhibitory isoform (LAP, 38 kD or LIP, 21 kDa, respectively) [20], [24]. Treatment of HT-29 cells with insulin for 24 h increased the nuclear levels of C/EBP alpha (isoform 42 kDa), of both C/EBP beta isoforms LAP and LIP, and decreased the nuclear levels of C/EBP alpha (isoform 30 kDa) in a dose-dependent manner. In parallel the expression of HSD11B2 decreased concomitantly with a maximal effect obtained at 10^−6^ M of insulin (Fig. 5A). However, in response to the same dose of insulin, the increase in LIP (≈130 fold at 10^−6^ M insulin) was greater than that in LAP (≈3 fold at 10^−6^ M insulin), resulting in a decreasing LAP/LIP ratio (Fig. 5A). Expression of C/EBP alpha (isoform 42 kDa) was slightly increased while the expression of C/EBP alpha (isoform 30 kDa) was decreased by 50% (Fig. 5A).

### HSD11B2 gene expression is up-regulated by C/EBP alpha/beta silencing

The effect of C/EBP alpha/beta knockdown on HSD11B2 was assessed in HT-29 cells. There is evidence from this siRNA transfection experiment that C/EBP alpha and C/EBP beta mRNA was downregulated significantly (Fig. 5C, D, left panel). Importantly, the mRNA levels of HSD11B2 increased following transfection with siRNA against both isoforms (Fig. 5C, D, right panel).

### Insulin regulation of C/EBP-DNA complexes

**Figure 6:**
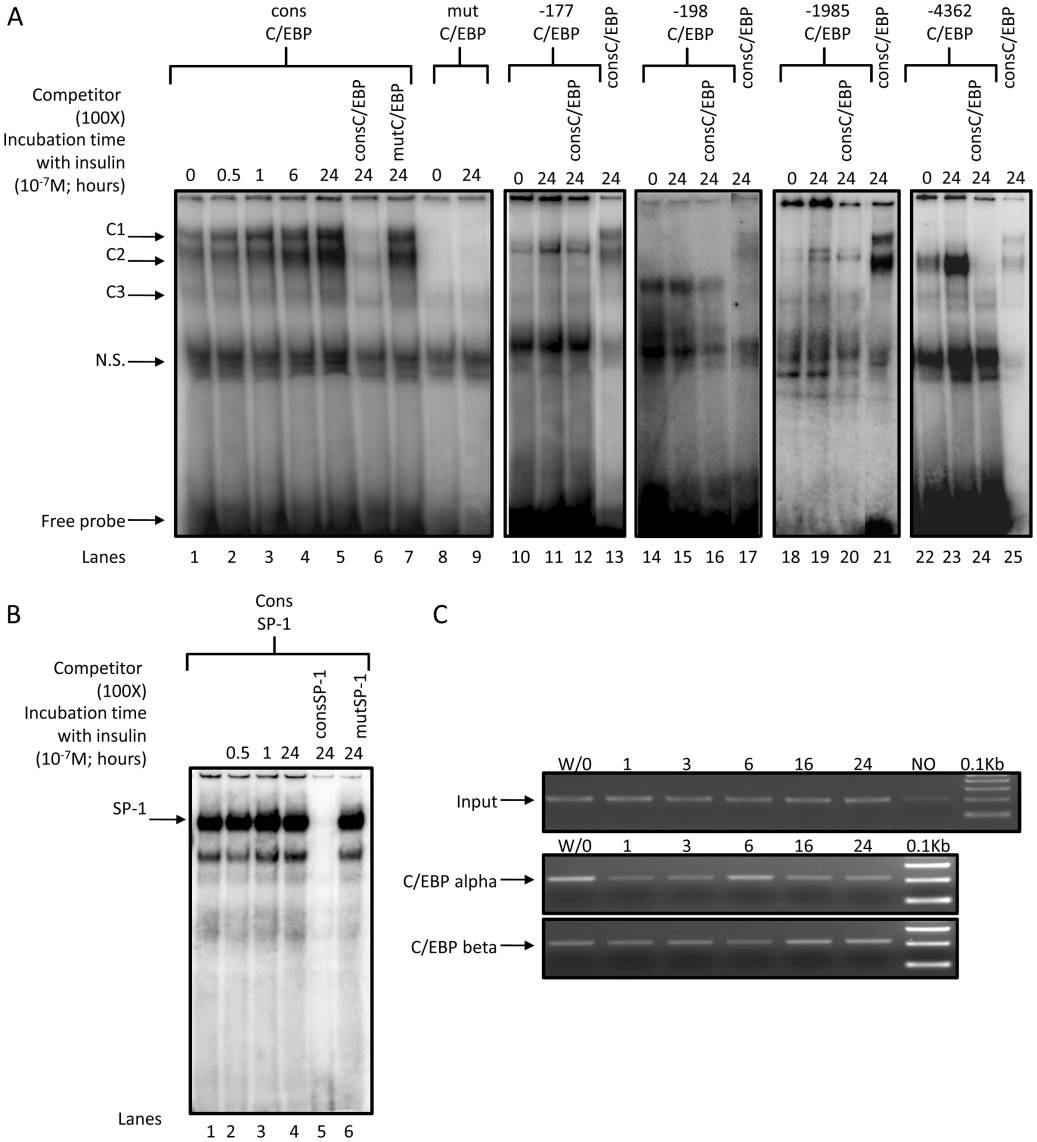
Binding of C/EBP alpha/beta on human HSD11B2 promoter. *(A) Nuclear proteins isolated from HT-29 cells bind to identified C/EBP alpha/beta sites.*4 µg of nuclear extracts isolated from insulin treated (for the indicated period of time, 10^−7^ M) or untreated HT-29 cells were incubated with radiolabeled probe encompassing the consensus C/EBP alpha/beta site in the presence or absence of non-radiolabeled (100×) competitor probe (cons C/EBP alpha/beta or mut C/EBP alpha/beta) (lanes1–7). Arrows indicate C/EBP alpha/beta / DNA shifts (C1, C2, C3) separated from free probe by gel electrophoresis. The complex C3 is formed in presence of radiolabeled −198 C/EBP alpha/beta probe (lanes 14–17) while complex C2 is formed in presence of radiolabeled −4362 C/EBP alpha/beta probe (lane 22–25). *(B) Nuclear proteins isolated from HT-29 cells bind to the consensus SP1 site.* Nuclear extracts isolated from insulin treated (for the indicated period of time, 10^−7^ M) or untreated HT-29 cells were incubated with radiolabeled probe encompassing the consensus SP1 site with and without non-radiolabeled (100X) competitor probe (cons SP1, lane 5or mut SP1, lane 6). The arrow indicates SP1/DNA shifts separated from free probe by gel electrophoresis. The complex intensity increased modestly with insulin treatment. The specific shift was abolished by the cold cons SP1 probe (lane 5) while not affected when mut SP1 probe was used as competitor (lane 6). *(C) Chromatin immunoprecipitation (ChIP) analysis of C/EBP alpha and C/EBP beta during insulin stimulation in HT-29 cells.* ChIPs were performed from untreated (W/O) and insulin induced (1–24 h) HT-29 cells using antibodies specific for C/EBP alpha (middle panel) and C/EBP beta (bottom panel), a no-antibody control (NO). The precipitated chromatin was analyzed using primers specific for the human HSD11B2 promoter. The DNA fragments were amplified with PCR primers to detect a 210 bp fragment containing the potential −177 and −198 C/EBP sites within the HSD11B2 promoter. Input chromatin is represented in upper panel.

The *in silico* analysis of the human HSD11B2 gene promoter sequence revealed 4 putative binding sites for C/EBPs located at positions −4361, −1985, −198 and −177 bp from the transcriptional start site (Table 1). The site −4361 has the higher match with the consensus sequence (Table 1). Different probes were labeled and incubated in presence of nuclear extracts isolated from insulin treated HT-29 cells. EMSA performed with the probe containing the consensus C/EBP binding site revealed three specific complexes (Fig. 6A, lane 1, noted C1-3). The signals were reversed by competition with the unlabelled probe harboring the consensus C/EBP site (Fig. 6A, lane 6) while unaffected when the probe harbored the mutated C/EBP sites (Fig. 6A, lane 7). Therefore, C1-3 signals might correspond to C/EBP/DNA complexes. The C/EBP binding to the consensus probe was elevated with increased duration of insulin treatment (Fig. 6A, lanes 1–5) reflecting the increased level of C/EBP beta found by Western Blot (Fig. 5A). Interestingly, the intensity of C2 increased more than C1, C2 being more abundant relatively to C1 24 h after insulin treatment than in controls (lanes 1, 5).

**Table 1:** Probes used for the EMSA experiments with C/EBP.

Matrix C/EBP	A_(G/C)_T_(A)_T_(G/A)_G_(A/T)_ C_(G/A)_ G_(C/A)_C_(A/T)_A_(C)_AT_(G/A)_	
cons C/EBP	5′-tgcag**ATTGCGCAAT**ctgca-3′	(100%)
mut C/EBP	5′-tgcag**A** GACTAGTC **T**ctgca-3′	(20%)
−177 C/EBP	5′-tccggctT **TT**T**C**CA**AAT**cgaatct-3′	(60%)
−198 C/EBP	5′-aaC**TT**TG**G** G **A** C **T**ttgttccg-3′	(50%)
−1985 C/EBP	5′-tcctgC**TT**TA**GCAA**Gtgctg-3′	(60%)
−4361 C/EBP	5′-gagagC**TTG**A**GCAAT**tccct-3′	(80%)

The weight matrix for the consensus C/EBP alpha/beta binding motif is given on top. The consensus C/EBP alpha/beta binding motif was aligned with the potential C/EBP binding sites identified in the human HSD11B2 promoter and located at position −177, −198, −1985, −4362 bp. “cons C/EBP” and “mut C/EBP” designate 20 to 24-mer oligonucleotides based respectively on the consensus and mutated binding site for C/EBP. Mismatched nucleotides with matrix are underlined. In bold are the nucleotides identical to the consensus sequence and the percentage of match with the consensus sequence is indicated.−177 C/EBP, −198 C/EBP, −1985 C/EBP,−4362 C/EBP indicate the probes harboring the putative binding sites for C/EBP alpha/beta located in the human HSD11B2 promoter.

The probes −198 and −4361 (Table 1) elicited the formation of complexes C2 or C3, but lacked C1 (Fig. 6A, lanes 14, 22). Complex formation were increased with insulin, albeit modestly for −198 (Fig. 6A, lanes 15, 23), and were reversed by competition with the probe harboring the consensus C/EBP site (Fig. 6A, lanes 16, 24). The complexes formed with −177 and −1985 were weak and not specific.

The binding of SP1, a transactivating factor known to regulate HSD11B2 expression [25], to its consensus binding site (Table 2) occurred in the unstimulated condition and was slightly increased upon insulin treatment (Fig. 6B, lane 1–4), suggesting that this factor might not be involved in the HSD11B2 repression upon insulin stimulation.

**Table 2:** Probes used for the EMSA experiments with SP1.

Matrix SP-1	G_(A/T)_G_(A)_G G C_(A/T)_G G G_(A/C)_	
cons SP-1	5′-attcgatc**GGGGCGGG**gcgagc-3′	(100%)
mut SP-1	5′-attcgatc**GG** TT **CGGG**gcgagc-3′	(80%)

The weight matrix for the consensus SP1 binding motif is given on top. “cons SP1” and “mut SP1” designate 22-mer oligonucleotides based respectively on the consensus and mutated binding site for SP1. Mismatched nucleotides with matrix are underlined. In bold are the nucleotides identical to the consensus sequence and the percentage of match with the consensus sequence is indicated.

Considering the original pattern of the complexes formed with the probe −198, a ChIP assay was performed, which actually confirms the binding of CEBP isoforms. Upon insulin treatment, CEBP beta binding to HSD11B2 promoter increased in a time dependent manner, while CEBP alpha interaction decreased (Fig. 6C), in agreement with the level of respective proteins (Fig. 5A).

### Modulation of HSD11B2 promoter activity by C/EBP beta isoforms

**Figure 7:**
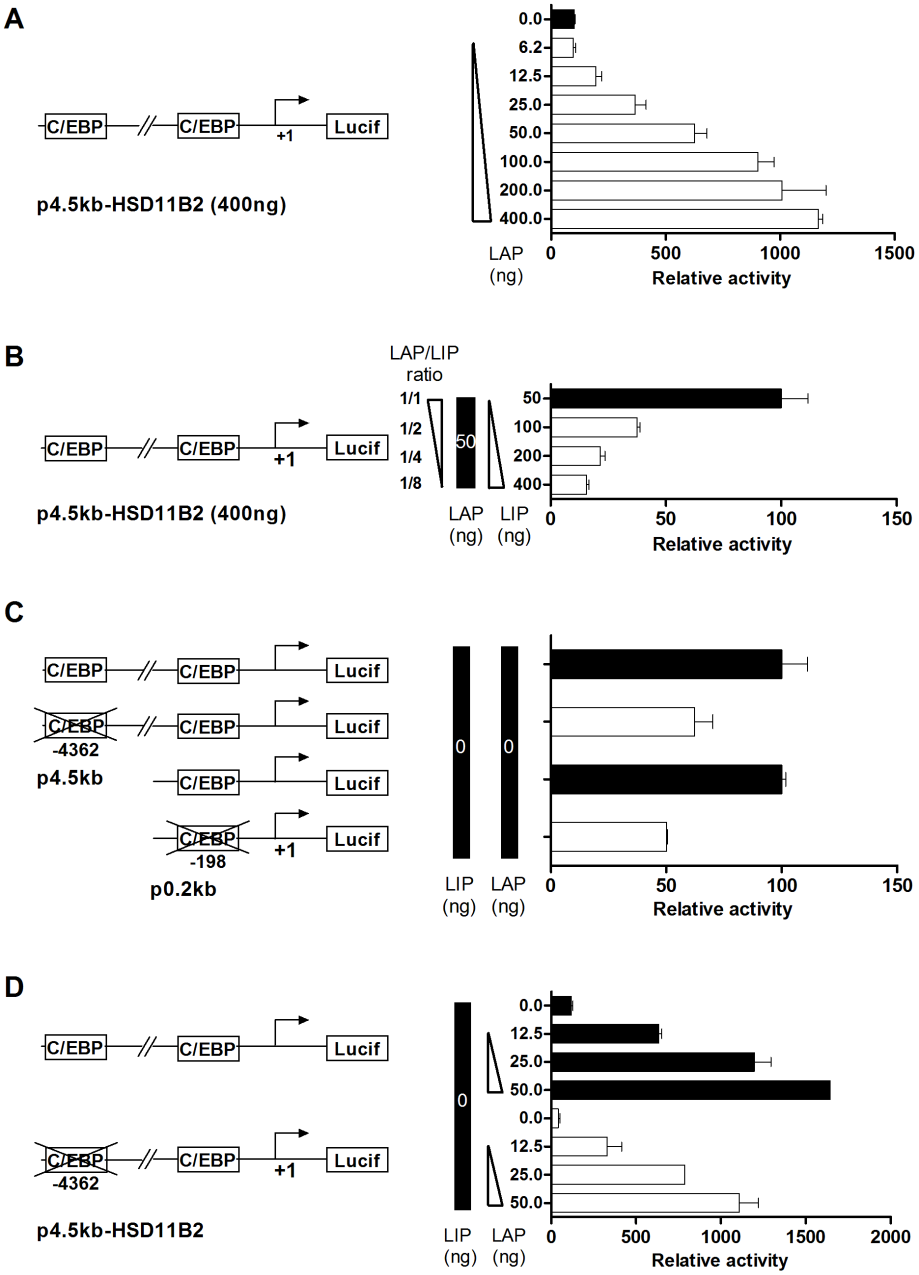
C/EBP beta isoforms control HSD11B2 promoter activity. (*A*) HT-29 cells were transfected with the full length human HSD11B2 promoter cloned into pGL3-basic luciferase vector (p4.5 kb-HSD11B2, 400 ng) and a dose response of LAP expressing vector (pCMV-LAP, 6.25 to 400 ng). A schematic representation of the promoter of HSD11B2 is shown on the left side. The transcriptional initiation site is indicated by an arrow (+1). The empty pcDNA3 vector was used to equalize the amount of transfected DNA in every condition and the pCMV-hRL (100 ng) was used as transfection efficiency control. Cells were lysed for luciferase assays 24 h after transfection, and the reading were normalized by renilla activity. (*B*) HT-29 cells were transfected with the plasmids p4.5 kb-HSD11B2 (400 ng), pRL-CMV (100 ng), pCMV-LAP (50 ng) and an increasing quantity of pCMV-LIP (50 ng to 400 ng). (*C*) HT-29 cells were transfected with the wild type p4.5 kb-HSD11B2 and p0.2 kb-HSD11B2 constructs or with the C/EBP mutated constructs. (*D*) HT-29 cells were transfected with the wild type p4.5 kb-HSD11B2 or the C/EBP mutated construct together with increasing concentration of pCMV-LAP.

To confirm the importance of the LAP/LIP ratio in the regulation of HSD11B2 gene expression at the transcriptional level, we used a reporter assay (Fig. 7). The construct p4.5 kb-HSD11B2 encompasses the region −4.5 kb to +0.116 kb of the human HSD11B2 promoter cloned in front of the luciferase encoding plasmid pGL3. Luciferase activity was measured as an indicator of HSD11B2 promoter activity. The construct, p4.5 kb-HSD11B2 was co-transfected into HT-29 cells with the plasmids encoding the long isoform of C/EBP beta (LAP) alone or in combination with the short isoform (LIP). We used the pcDNA-LAP/pcDNA-LIP plasmid DNA ratio to represent the LAP/LIP ratio in transfected cells, while keeping the total amount of plasmid transfected constant (pcDNA3 empty vector was used to compensate DNA quantities). The luciferase activity correlated with the amount of pcDNA-LAP transfected (Fig. 7A). In contrast, increasing LIP expression decreased luciferase activity (Fig. 7B). To validate the role of characterized C/EBP beta binding sites, mutagenesis was performed. By mutating the sites −4392 and −198, the basal (Fig 7C) and the LAP (Fig 7D) induced promoter activities were partly reduced. This data suggested that several binding sites participated in the C/EBP mediated HSD11B2 promoter activity. Surprisingly, the reporter assay experiments failed to show any insulin-dependent regulation of HSD11B2 promoter, suggesting that insulin action might be mediated at an epigenetic level.

### The insulin-dependent lactate synthesis modulated 11beta-HSD2 activity

**Figure 8:**
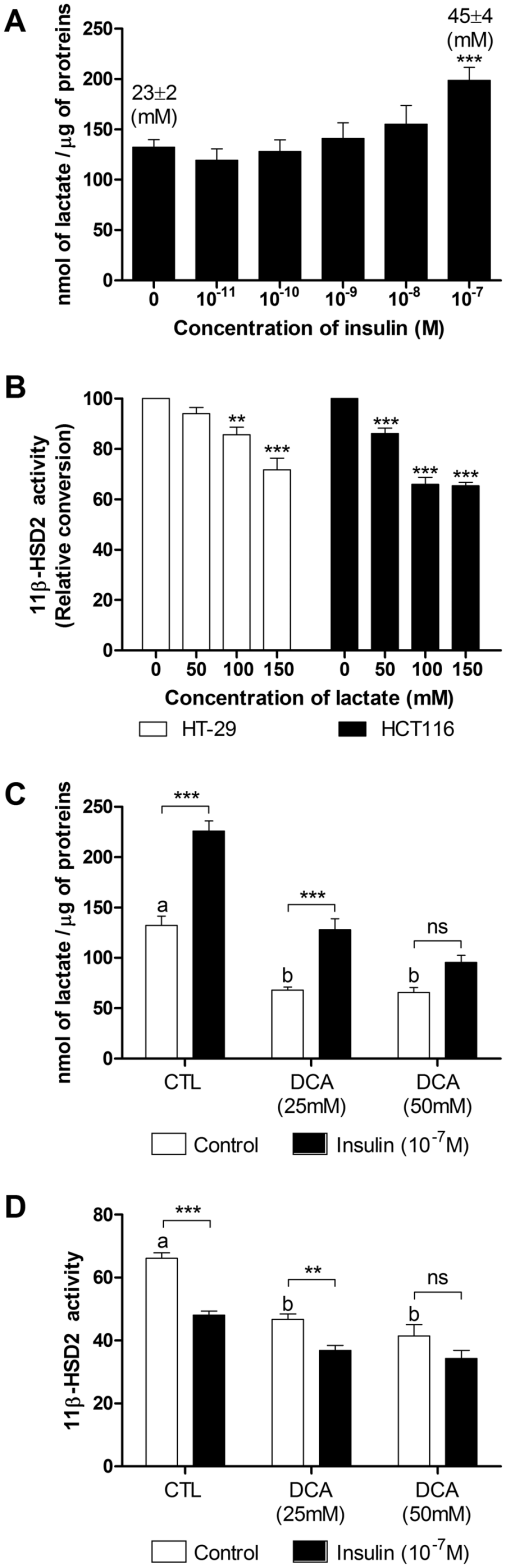
Lactate accumulation in the media upon insulin stimulation and insulin-dependent down-regulation of 11beta-HSD2 activity. (*A*) Dose-response effect of insulin on L-lactate production in cultured HT-29 cells after 24 h incubation. The concentration in lactate found in the media of HT-29 cells after 24 h of culture is reported above the bars (Mean +/− SEM). (*B*) 11beta-HSD2 activity in cultured HT-29 and HCT116 cells exposed to exogenous L-lactate for 3 h. (*C*) 24 h L-lactate production in cultured HT-29 cells exposed to DCA alone or in combination with insulin. (*D*) 11beta-HSD2 activity in cultured HT-29 cells exposed to DCA alone or in combination with insulin.

Next, we challenged the hypothesis that lactate, a potential HDAC inhibitor and a byproduct of glycolysis, which is increased under insulin stimulation mediates HSD11B2 downregulation. Lactate secretion was quantified under insulin treatment and 11beta-HSD2 activity monitored under lactate stimulation or lactate synthesis blockage. Figure 8A shows a dose dependent increase in lactate secretion by insulin in HT-29 cells. Treatment with lactate alone significantly reduced 11beta-HSD2 activity in HT-29 and HCT116 cells (Fig. 8B). Dichloroacetate (DCA) is a pyruvate dehydrogenase kinase (PDK) inhibitor, whose action restores the normal oxidative demolition of pyruvate and thus indirectly preventing glycolysis [26]. Used alone, DCA reduced lactate production in HT-29 (Fig. 8C) however, in combination with insulin, it reduced insulin-dependent stimulation of lactate secretion (Fig. 8C). Most importantly, DCA reduced insulin-dependent downregulation of 11beta-HSD2 activity (Fig. 8D).

## Discussion

### Insulin-dependent regulation of HSD11B2

The present investigation revealed in three different human cell lines, that insulin reduces the activity of 11beta-HSD2. We report for the first time, that the dose- and time-dependent effect of insulin is attributable to diminished transcriptional activity, as opposed to the stability of the transcribed mRNA. A peculiar finding of the insulin-induced down-regulation of HSD11B2 is the increase in mRNA levels during the first 8–10 h, without a concomitant increase in the activity or protein content (Fig. 1C), an observation previously made for C/EBPs. The mechanism for this discrepancy is unknown. One possible explanation might be the temporal induction of small regulatory RNA molecules, interfering with transcription, as it has recently been demonstrated for GLUT-4, hormone sensitive lipase, fatty acid-binding protein ap2 and peroxisome proliferator-activated receptor gamma 2 genes [27], [28].

### Mechanisms accounting for insulin-dependent HSD11B2 downregulation

Our study suggests that an insulin-dependent decrease in HSD11B2 expression could be related to changes in the LAP/LIP ratio, chromatin structural changes or lactate production.

#### 1-Considering decreased LAP/LIP ratio to inhibit HSD11B2 expression

An *in silico* analysis of the HSD11B2 promoter predicted binding-sites for C/EBPs. This is important since insulin is known to modulate the expression of two isoforms of C/EBP beta, LAP and LIP [10], [11], [23], [29]. LAP/LIP ratio is modulated by mTOR, a downstream target of the insulin pathway, shifting C/EBP translation toward LIP translation [24]. We made the interesting observation that mTOR and AKT VIII inhibitor rescued HSD11B2 expression. Moreover, EMSA experiments demonstrated that following insulin stimulation, there was an increased association of C2 product to the HSD11B2 promoter. According to the literature this C2 product comprises a LAP/LIP dimer [20], [30]. These correlations were ascertained by reporter assays showing i) an up-regulation of the promoter activity concomitant with LAP overexpression, ii) the requirement of both non-canonical C/EBP binding sites for the promoter activity, and iii) the sensitivity of the reporter construct towards the C/EBP beta LAP/LIP ratio. Taken together, the data suggest that C/EBP beta, most probably LAP, regulates the basal expression of HSD11B2, while LIP mediates insulin dependent HSD11B2 gene repression. Hence, HSD11B2 expression is regulated by LAP/LIP ratio in a way similar to HSD11B1 [12], [14].

#### 2-Other potential participants for an insulin-dependent inhibition of HSD11B2 transcription

Despite the important findings concerning the regulatory role of the LIP/LAP ratio, some questions still remain in order to understand the mechanism of the insulin-dependent decrease of the HSD11B2 expression. In transfected, cells we observed the inability of insulin to downregulate the expression of reporter gene fused to the HSD11B2 promoter (data not shown). We first hypothesized that by transfecting a large amount of plasmid into the cells, the number of *cis* elements available for C/EBP proteins are far in excess. In this scenario, the newly synthesized LIP molecules in presence of insulin had the ability to bind plasmidic DNA without displacement of the bound LAP.

Because HSD11B2 transcription is activated in the first hours and inhibited in the last hours of insulin treatment, it might be possible that the stability of the luciferase protein did not reflect the real time activity of the promoter. Indeed, highly stable reporters accumulate to greater levels in cells, but their concentrations change slower relative to changes in transcription. Additional experiments, in which the promoter of HSD11B2 is cloned into a plasmid encoding for an unstable reporter gene, including for example a PEST signal, would challenge this hypothesis.

Moreover, gene repression is sometimes dependent on chromosome-embedment and additional sequences located either far away from the promoter in 5′ as described for the PEPCK gene promoter under insulin treatment [31], or even within the 3′ region in the intronic sequence might account for the insulin-dependent downregulation of HSD11B2. A sequence alignment using the VISTA program shows some sequences well conserved in intron I that could potentially act as intronic enhancers (Fig. S2) [32].

Furthermore, gene expression is also regulated by histones and DNA wrapping. Yet, transiently transfected DNA acquires a conformation, structurally different for the counterpart chromatin integrated DNA that may underlie the differences in the mechanisms of activation of the two templates [33]. Hence, we cannot exclude that epigenetic mechanisms (i.e. histone deacetylation and DNA methylation) are involved in the insulin-dependent HSD11B2 downregulation. In line with this, HSD11B2 gene contains 2 CpG islands within the promoter that indeed regulate gene expression [34]. Moreover, C/EBP beta is known to cooperate with coactivators such as SWI/SNF which only work in chromosome-embedded gene [35].

#### 3-The potential role of lactate production to inhibit HSD11B2 transcription

Interestingly, mRNA profiling underlined the reprogramming of the transcriptome from insulin sensitive cells towards insulin insensitive cells, with activation of the glycolytic pathway and consequently lactate production (Table S1, Fig. 4). In line with the literature, lactate secretion and pH changes were monitored in HT-29 cells upon insulin treatment [36]. On one hand, a decrease in pH was shown to inhibit 11beta-HSD2 activity in kidney tubules directly [37], while on the other hand, lactate was shown to inhibit HDAC activity directly and by this fact to regulate gene expression in HCT116 [38]. The 153 gene probes, including HSD11B2, down-regulated by all four HDAC inhibitors are listed (Supplementary Table 2 of [38]). In agreement with this observation, an inhibition of lactate synthesis by DCA reduced significantly the insulin effect, while treatment with lactate repressed 11beta-HSD2 activity in our cellular models (HT-29 and HCT116). In this respect, lactate can be considered to be a potential regulator of HSD11B2 expression, independently or in parallel to LIP/LAP. This fact is also strengthened by our previous observation of a decreased HSD11B2 expression along the rat intestine [39], which is inversely correlated with the intestinal lactate concentration [40]. Lactate is produced by bacteria of the gut and is found in the rectum in a millimolar range, when physiological situations are considered [41]. Our finding that 11beta-HSD2 activity was decreased using 50mM lactate, is consistent with the literature [38], although, it is uncertain if such concentrations could be reached locally in the gut or if such a down regulation would happen in vivo with longer exposure and lower amounts. Nevertheless, an increase in the abundance in lactic acid bacteria, associated with methylation changes in intestinal cells was reported in type 2 diabetic patients [42]. Moreover, plasmatic lactate is associated with blood pressure [43] and type 2 diabetes [44]. In addition, lactate is also produced (up to 17 mM) during ischemia in kidneys [45]. Notably ischemia was related to renal tubular dysfunctions with increased blood pressure and reduced 11beta-HSD2 activity [46]. Finally, lactate, as an indicator of oxidative capacity, was found to predict incident diabetes, since oxidative capacity is decreased in type 2 diabetes. Lactate is therefore strongly related to insulin resistance [47]. Whether decreased oxidative capacity is a cause or consequence of diabetes is unknown, but the link with HSD11B2 downregulation has to be strongly considered.

Sodium reabsorption is an important function of the kidney, but also of the rectal and colonic mucosa [48], [49]. This mechanism is regulated, at least in part, by MR [50], with subsequent activation of the the amiloride-sensitive epithelial sodium channel (ENaC) [51], [52]. Here, we provide evidence for an insulin-dependent downregulation of HSD11B2, a prerequisite for cortisol-mediated MR transactivation leading to an increase in sodium reabsorption in the colon. All together, these data suggest that the downregulation of HSD11B2 expression in cancer colonic cell lines, after long-term insulin treatment would be the consequence of LIP overexpression, together with increased lactate production, both working at an epigenetic level. These mechanisms are of interest and significance for the understanding sodium reabsorption in the colon in health and disease states.

## Supporting Information

Figure S1 **Sustained insulin treatment diminished the 11beta-HSD2 expression and actiity in JEG-3 cells.** (*A*) 11beta-hydroxysteroid dehydrogenase type 2 (HSD11B2) expression was assessed by qRTPCR in SW620 (gray bars), JEG-3 (hatched bars), and HT-29 (filled bars) cells 24 h after incubation with insulin (10^−7^ M). (*B*) 11beta-HSD2 activity was measured by ^3^H-Cortisol/Cortisone conversion assay in SW620, JEG-3, and HT-29 cells 24 h after incubation with insulin (10^−7^ M).
(TIF)

Figure S2 **Sequence homology between human and rat HSD11B2 genes.** Upper part, representation of HSD11B2 gene with the exons boxed in violet and the untranslated region boxed in light green. Lower part, percentage of homology between human and rat sequences. Regions with more than 80% homology are boxed in green and noted with a or b.
(TIF)

Table S1 **Transcriptional regulation of insulin pathway related genes in HT-29 cells by sustained insulin stimulation.**
(DOCX)
