# Supplementary figures and images for: Insulin, CCAAT/Enhancer-Binding Proteins and Lactate Regulate the Human 11β-Hydroxysteroid Dehydrogenase Type 2 Gene Expression in Colon Cancer Cell Lines
Source: PLoS One. 2014 Aug 18;9(8):e105354. doi: 10.1371/journal.pone.0105354 (PMC4136812; doi:10.1371/journal.pone.0105354)

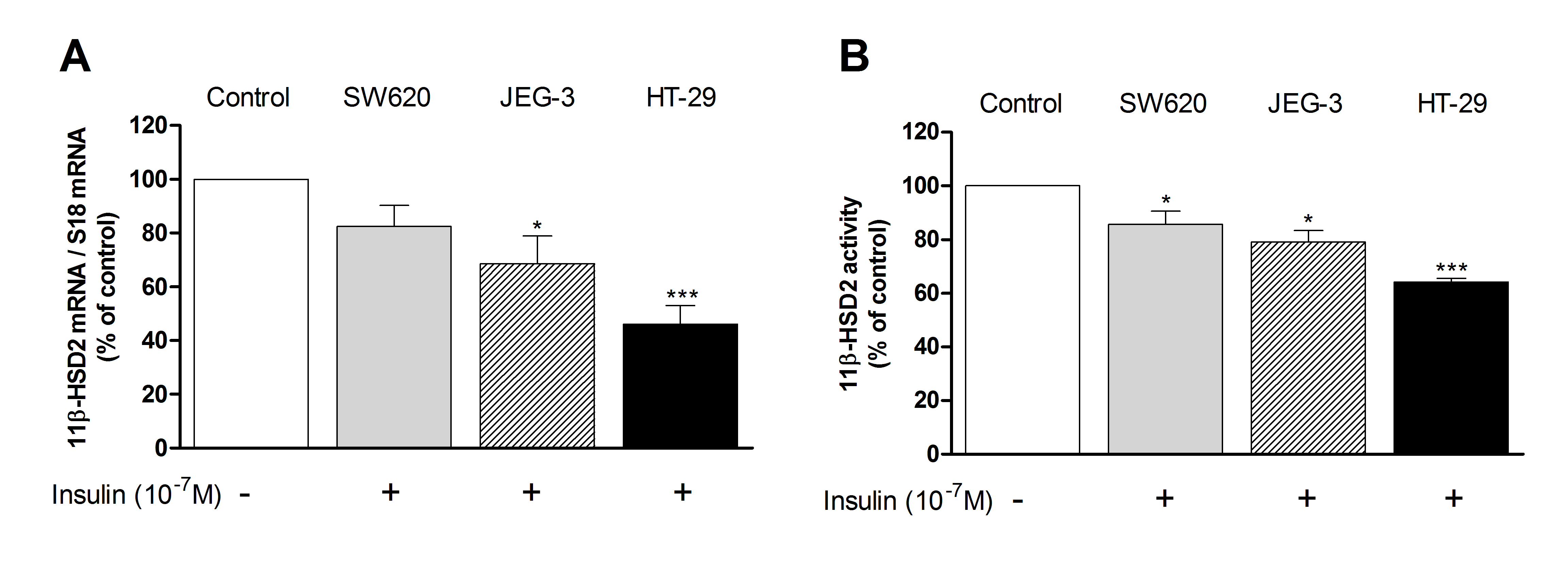

Supplement: Figure S1 — Sustained insulin treatment diminished the 11beta-HSD2 expression and actiity in JEG-3 cells. (A) 11beta-hydroxysteroid dehydrogenase type 2 (HSD11B2) expression was assessed by qRTPCR in SW620 (gray bars), JEG-3 (hatched bars), and HT-29 (filled bars) cells 24 h after incubation with insulin (10−7 M). (B) 11beta-HSD2 activity was measured by 3H-Cortisol/Cortisone conversion assay in SW620, JEG-3, and HT-29 cells 24 h after incubation with insulin (10−7 M). (TIF) [file pone.0105354.s001.tif]

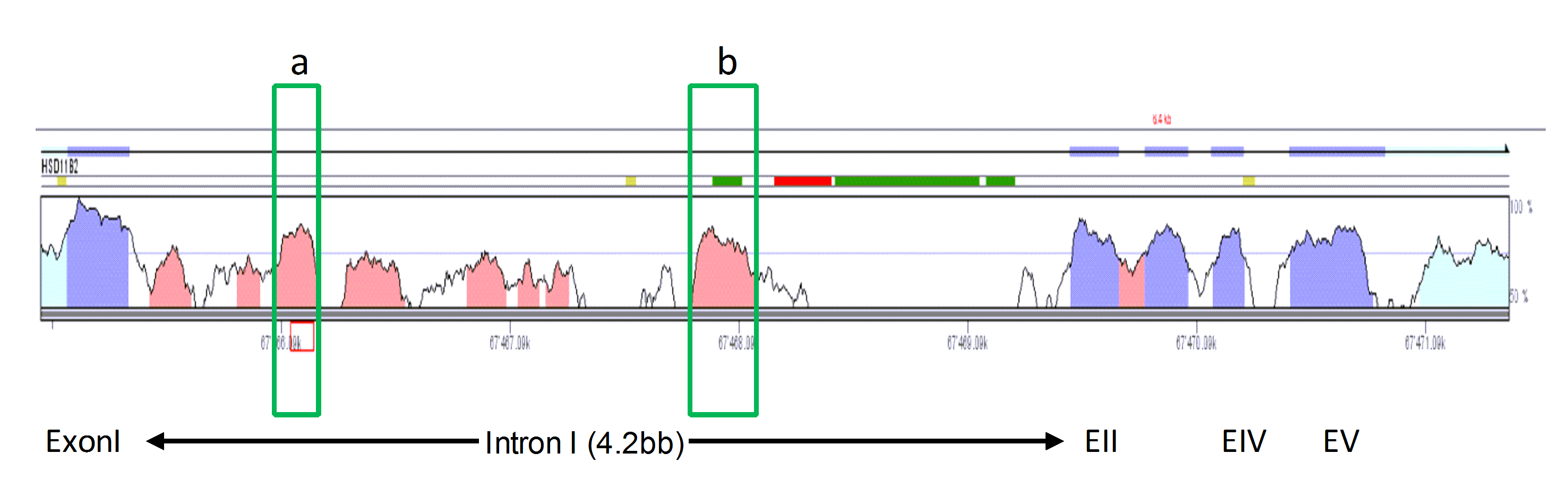

Supplement: Figure S2 — Sequence homology between human and rat HSD11B2 genes. Upper part, representation of HSD11B2 gene with the exons boxed in violet and the untranslated region boxed in light green. Lower part, percentage of homology between human and rat sequences. Regions with more than 80% homology are boxed in green and noted with a or b. (TIF) [file pone.0105354.s002.tif]
