# Supplementary material for: Insulin, CCAAT/Enhancer-Binding Proteins and Lactate Regulate the Human 11β-Hydroxysteroid Dehydrogenase Type 2 Gene Expression in Colon Cancer Cell Lines
Source: PLoS One. 2014 Aug 18;9(8):e105354. doi: 10.1371/journal.pone.0105354 (PMC4136812; doi:10.1371/journal.pone.0105354)
Supplement: Table S1 — Transcriptional regulation of insulin pathway related genes in HT-29 cells by sustained insulin stimulation. (DOCX) [file pone.0105354.s003.docx]

**Table S1:**

| **A-Upregulated genes** | |  |  |
| --- | --- | --- | --- |
| **Symbol** | **Gene bank** | **Description** | **Fold change** |
| MAP2K1 | NM_002755 | Mitogen-activated protein kinase kinase 1 | **2.85** |
| SLC2A1 | NM_006516 | Solute carrier family 2, member 1 | **2.02** |
| PKM2 | NM_002654 | Pyruvate kinase, muscle | 1.82 |
| IGFBP1 | NM_000596 | Insulin-like growth factor binding protein 1 | 1.54 |
| EIF4EBP1 | NM_004095 | Eukaryotic translation initiation factor 4E binding protein 1 | 1.50 |
| HK2 | NM_000189 | Hexokinase 2 | 1.46 |
| CEBPB | NM_005194 | CCAAT/enhancer binding protein (C/EBP), beta | 1.25 |

| **B-Downregulated genes** | | | |
| --- | --- | --- | --- |
| **Symbol** | **Gene bank** | **Description** | **Fold change** |
| GAB1 | NM_002039 | GRB2-associated binding protein 1 | -1.46 |
| FOS | NM_005252 | V-fos FBJ murine osteosarcoma viral oncogene homolog | -1.48 |
| ANG | NM_001145 | Angiogenin, ribonuclease, RNase A family, 5 | -1.54 |
| SLC2A4 | NM_001042 | Solute carrier family 2 (facilitated glucose transporter), member 4 | -1.55 |
| GPD1 | NM_005276 | Glycerol-3-phosphate dehydrogenase 1 (soluble) | -1.60 |
| BRAF | NM_004333 | V-raf murine sarcoma viral oncogene homolog B1 | -1.68 |
| SORBS1 | NM_006434 | Sorbin and SH3 domain containing 1 | -1.71 |
| IGF1R | NM_000875 | Insulin-like growth factor 1 receptor | -1.73 |
| ACOX1 | NM_004035 | Acyl-Coenzyme A oxidase 1, palmitoyl | -1.80 |
| CEBPA | NM_004364 | CCAAT/enhancer binding protein (C/EBP), alpha | -1.84 |
| AEBP1 | NM_001129 | AE binding protein 1 | -1.97 |
| FBP1 | NM_000507 | Fructose-1,6-bisphosphatase 1 | **-2.28** |
| IGF2 | NM_000612 | Insulin-like growth factor 2 (somatomedin A) | ***-2.50*** |
| IRS2 | NM_003749 | Insulin receptor substrate 2 | **-3.02** |
| INSR | NM_000208 | Insulin receptor | **-3.21** |
